# Determinants and typologies of health system resilience: A data-driven framework for prioritising investment

**DOI:** 10.1371/journal.pone.0340560

**Published:** 2026-09-23

**Authors:** Richmond Balinia Adda, Conrad Pwayirane

**Affiliations:** 1 Department of Biometry, School of Mathematical Sciences, University of Technology and Applied Sciences, Navrongo, Ghana; 2 Department of Epidemiology and Biostatistics, School of Public Health, University of Technology and Applied Sciences, Navrongo, Ghana; USTC: University of Science and Technology of China, CHINA

## Abstract

Health system resilience is essential for effective pandemic response and health security, yet existing assessments often lack actionable guidance for policymakers. This study develops a data-driven framework to identify key resilience contributors and country capability profiles using the complete 2021 Global Health Security Index (GHSI) dataset for 195 countries. Through a multi-method analytical approach, we derive a composite resilience measure that captures 70.6% of shared variance across six GHSI domains. After accounting for inter-domain correlations, Detection, Health Capacity, and Prevention show the strongest independent associations with overall resilience (ridge coefficients of 0.242, 0.228, and 0.219, respectively), with Detection contributing most. Cluster analysis reveals two distinct country typologies: a high-performing, balanced-capability group (33% of countries) spanning both high-income and several upper-middle-income countries, and a larger group (67%) with lower and more structurally uneven capabilities, particularly in Prevention and Health Capacity. These profiles suggest different strategic pathways for system strengthening—from sustaining advanced capacities in high-performing countries to targeted investments in detection and core health functions in settings with more fragmented capabilities. Our findings provide an essential framework for comparing resilience across countries and prioritising investments where they matter most, offering practical guidance for national and global health security planning.

## 1 Introduction

Health system resilience has emerged as a critical determinant of national health security, as starkly demonstrated by the COVID-19 pandemic and its uneven impact across countries [1]. Beyond static measures of preparedness, resilience reflects a system’s ability to absorb shocks, adapt to changing threats, and maintain essential services during crises [2,3]. Countries with more resilient health systems have consistently experienced lower excess mortality and better continuity of care during emergencies [4,5]. Yet translating this recognition into actionable policy remains challenging, particularly given the multidimensional nature of resilience and varying national contexts.

The 2021 Global Health Security Index (GHSI) provides one of the most comprehensive assessments of national health security capacities, spanning six domains: prevention, detection, response, health system capacity, commitments, and risk environment [6]. While widely referenced, the index is primarily descriptive and offers limited guidance on how domains interact to produce resilience or how countries with similar scores may differ in their underlying capability profiles. Existing analyses often focus on isolated components [7] rather than the systemic interdependencies that characterise resilient health systems [8].

This gap between measurement and action is particularly consequential for policymakers and health planners who must prioritise investments under resource constraints. A more nuanced, empirically grounded framework is needed—one that identifies not only which capabilities matter most for resilience but also how countries cluster into meaningful typologies with distinct strengthening pathways.

To address this need, we developed a multi‑method analytical framework using the 2021 GHSI data to derive a data‑driven resilience index, quantify the relative importance of different domains, and identify country clusters with similar capability patterns. Our approach integrates dimension reduction, uncertainty quantification, and pattern recognition techniques to provide a reproducible and policy‑relevant assessment. In particular, the study examines the underlying structure of health system resilience as measured by the GHSI and evaluates its robustness, investigates which domains contribute most strongly to overall resilience when accounting for their interrelationships, and explores the distinct country typologies that emerge based on resilience capabilities and their implications for targeted health system strengthening. The analysis is grounded in a complex adaptive systems perspective [9,10], which views resilience as an emergent property of interactions among governance, resources, service delivery, and external pressures. By combining principal component analysis with bootstrap validation, regularised regression, and cluster analysis, we provide an integrated assessment that reflects these interdependencies while offering concrete insights for decision‑makers.

The findings are intended to support strategic planning and investment prioritisation, especially in settings where resources are limited and targeted improvements can yield the greatest benefit [11]. The remainder of the paper is structured as follows: the Methods section describes the data and analytical approach; the Results section presents the resilience index, domain importance, and country typologies; the Discussion section examines policy implications, limitations, and directions for future research.

## 2 Methodology

### 2.1 Data source and variables

#### 2.1.1 Global health security index dataset.

This study uses the 2021 Global Health Security Index (GHSI) dataset [6]. The GHSI is compiled by the Nuclear Threat Initiative, the Johns Hopkins Center for Health Security, and Economist Impact from 304 underlying indicators for 195 countries, which the index’s publishers aggregate into six published domain-level scores: Prevention (P), Detection (D), Response (R), Health Capacity (H), Commitments (C), and Risk Environment (E), alongside an overall GHSI score. These domain-level scores, rather than the underlying indicators, are the analytical unit for this study — the PCA, bootstrap, ridge regression, and cluster analyses described below (Equations 1–9) all operate on the six domain scores per country.

For each country i=1,...,195, domain-level scores are denoted $P_{i}$, $D_{i}$, $R_{i}$, $H_{i}$, $C_{i}$, and $E_{i}$.

#### 2.1.2 Variable selection and preprocessing.

Countries with complete data across all domains were retained (*n* = 195). Domain scores were standardised to ensure comparability:


$$\begin{array}{c} Z_{ij}=\frac{X_{ij}-\mu_{j}}{\sigma_{j}}, \end{array}$$ (1)

where $Z_{ij}$ is the standardised score for country *i* on domain *j*, and $\mu_{j}$, $\sigma_{j}$ are the domain mean and standard deviation.

### 2.2 Ethical approval

This study uses publicly available, non-identifiable secondary data from the Global Health Security Index. No human participants were involved, and ethical approval was therefore not required.

### 2.3 Principal component analysis framework

#### 2.3.1 Rationale and theoretical background.

Principal Component Analysis (PCA) was used to derive a composite resilience index by summarising shared variation across domains [12–15]. PCA is appropriate here because the domains are highly correlated, and a lower-dimensional structure is expected. The eigenvalue problem is defined as:


$$\begin{array}{c} {Rv}_{k}=\lambda_{k}{𝐯}_{k}, \end{array}$$ (2)

where $\lambda_{k}$ and ${𝐯}_{k}$ are the eigenvalues and eigenvectors of the domain correlation matrix 𝐑.

#### 2.3.2 Resilience score construction.

The resilience score was defined as the first principal component:


$$\begin{array}{c} RS_{i}=\sum_{j=1}^{6}v_{1j}Z_{ij}, \end{array}$$ (3)

where *v*_1*j*_ are the loadings of the first eigenvector. This component captures the dominant pattern of variation across domains.

#### 2.3.3 Assumptions and limitations of PCA.

PCA assumes linear relationships among variables and relies on the covariance (or correlation) structure to identify components. Given the strong inter-domain correlations in the GHSI, PCA captures broad, system-wide capability patterns rather than isolated domain effects. However, PCA does not account for nonlinear interactions, may mix conceptually distinct domains, and does not impose theoretical constraints. These limitations were considered when interpreting components and when validating results with complementary methods.

### 2.4 Statistical validation framework

#### 2.4.1 Bootstrap uncertainty quantification.

Component uncertainty was evaluated using a non-parametric bootstrap with *B* = 500 resamples:


$$\begin{array}{c} RS_{i}^{\left(b\right)}=\sum_{j=1}^{6}v_{1j}^{\left(b\right)}Z_{ij}^{\left(b\right)}. \end{array}$$ (4)

The number of iterations was selected after examining the convergence of the first eigenvalue and loading distributions; stability improved minimally beyond 300 iterations, and results at *B* = 1000 did not differ meaningfully. Bootstrap percentile intervals were computed as:


$$\begin{array}{c} CI_{1-\alpha }(v_{1j})=\left[v_{1j}^{\left(\alpha /2\right)},v_{1j}^{\left(1-\alpha /2\right)}\right]. \end{array}$$ (5)

#### 2.4.2 Component stability.

Stability was assessed using the width of bootstrap-derived intervals:


$$\begin{array}{c} S_{j}=1-\left(v_{1j}^{\left(0.975\right)}-v_{1j}^{\left(0.025\right)}\right). \end{array}$$ (6)

Loadings with $S_{j}>0.5$ were considered stable.

### 2.5 Regularised regression analysis

#### 2.5.1 Addressing multicollinearity.

To estimate each domain’s unique contribution to overall performance, ridge regression was used:


$$\begin{array}{c} \min_{\beta }\{\|𝐘-𝐗\beta {\|}_{2}^{2}+\lambda \|\beta {\|}_{2}^{2}\}, \end{array}$$ (7)

where 𝐘 is the overall GHSI score and λ is selected via cross-validation. Ridge regression is suited for this analysis because the GHSI domains are strongly correlated, which inflates instability in ordinary least squares estimates [16,17].

The resulting *R*^2^ = 1.0 reflects the deterministic relationship between domain scores and the overall GHSI score, which is calculated directly from the domains. This does not indicate overfitting but follows from the index structure.

#### 2.5.2 Comparison with alternative methods.

Three measures of domain importance were compared:

1. Pearson correlations: $r_{j}=\text{corr}(X_{j},Y)$,
2. Ridge regression coefficients,
3. PCA loadings from the first component.

This triangulation provides a robust view of domain influence while accounting for multicollinearity.

### 2.6 Cluster analysis for resilience typologies

#### 2.6.1 Clustering procedure.

Country capability profiles were analysed using K-means clustering [18,19]:


$$\begin{array}{c} \min_{C_{1},...,C_{K}}\sum_{k=1}^{K}\sum_{i\in C_{k}}\|{𝐱}_{i}-\mu_{k}{\|}^{2}. \end{array}$$ (8)

#### 2.6.2 Cluster selection and interpretation.

The optimal number of clusters $K^{*}$ was chosen using the silhouette score [20]:


$$\begin{array}{c} s(i)=\frac{b(i)-a(i)}{\max\{a(i),b(i)\}}, \end{array}$$ (9)

where *a*(*i*) is intra-cluster distance and *b*(*i*) is the nearest-cluster distance. Silhouette values indicated moderate-to-good separation, suggesting that meaningful typologies exist while country capabilities also lie along a continuum. Clusters are therefore interpreted as profiles rather than rigid groups.

### 2.7 Methodological robustness checks

Sensitivity analyses assessed the stability of results by varying bootstrap iterations (*B* = 200, 500, 1000), testing alternative clustering algorithms (DBSCAN, hierarchical clustering), and altering ridge regularisation strengths. Two alternative resilience measures—a simple average and a correlation-weighted average—were compared to the PCA-based score to evaluate consistency [21,22].

### 2.8 Statistical software and implementation

All analyses were implemented in Python 3.9 using scikit-learn [23], SciPy, and Matplotlib. Custom scripts were developed for the bootstrap PCA and sensitivity analyses.

## 3 Results

### 3.1 Principal component analysis of health system resilience

#### 3.1.1 Variance decomposition.

The PCA results show a strong underlying structure in the health security domains. Table 1 reports the full variance decomposition, computed from the verified 2021 GHSI domain scores for all 195 countries. The first principal component (PC1) explains 70.63% of the total variance, indicating that a single dimension summarises most of the shared variation across domains.

**Table 1:** Variance decomposition in principal component analysis.

Component	Eigenvalue	Variance Explained (%)	Cumulative Variance (%)
PC1	4.2598	70.63	70.63
PC2	0.6666	11.05	81.68
PC3	0.4088	6.78	88.46
PC4	0.3836	6.36	94.82
PC5	0.1820	3.02	97.84
PC6	0.1302	2.16	100.00

**Fig 1:**
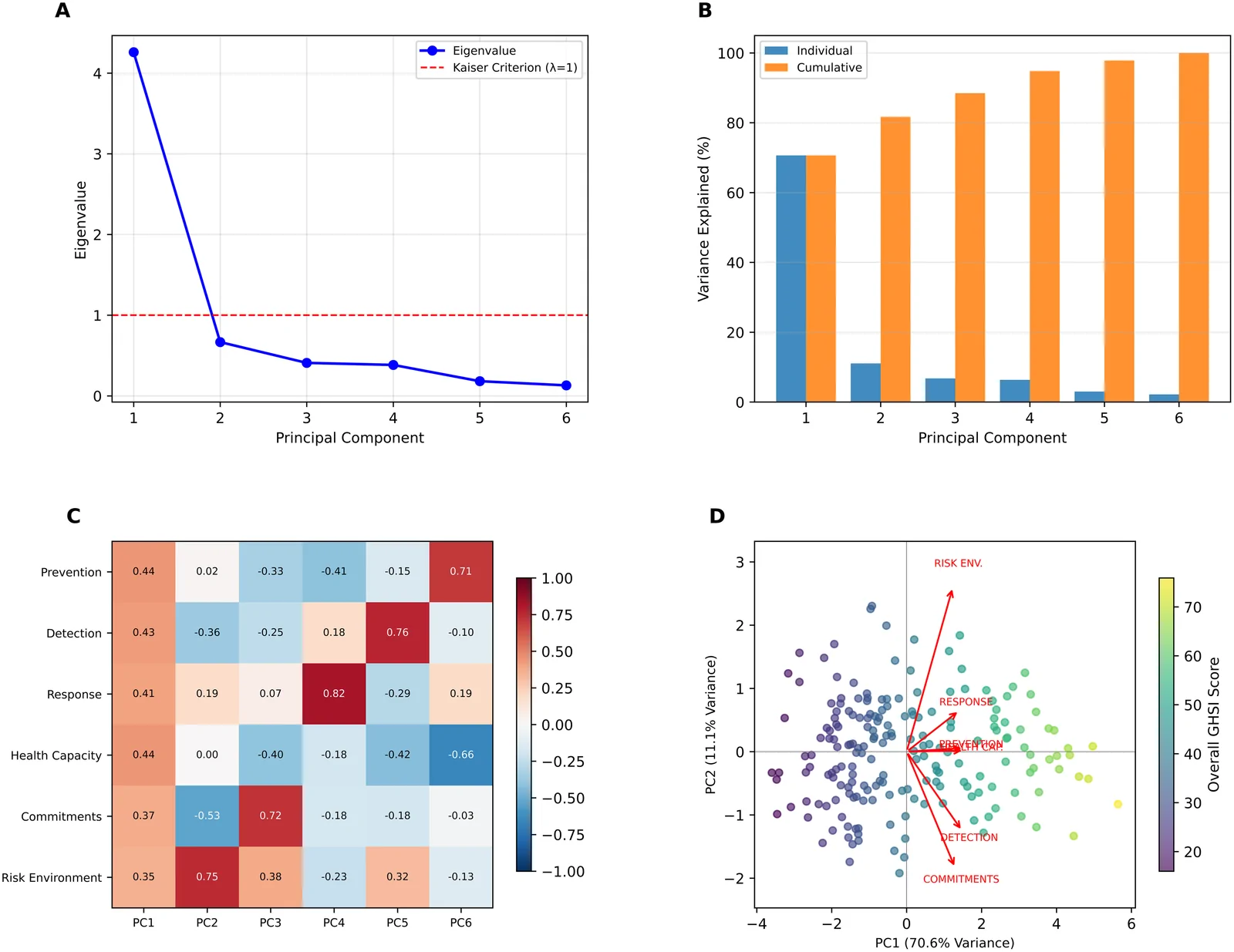
Visual summary of PCA results. Panel A: scree plot. Panel B: variance explained. Panel C: loading heatmap. Panel D: PC1–PC2 biplot.

The scree plot (Fig 1A) shows a clear drop after the first component, supporting the choice of PC1 as the summary resilience measure. The first two components together explain 81.68% of the total variance.

#### 3.1.2 Component loadings.

Table 2 presents the loadings for all components. The loadings for PC1 are positive and similar in size, with the largest contributions from Health Capacity (0.4436), Prevention (0.4402), and Detection (0.4260). This pattern suggests that PC1 reflects a general resilience dimension: countries that perform well in one domain tend to perform well in others.

**Table 2:** Component loadings (eigenvectors) for principal components.

Domain	PC1	PC2	PC3	PC4	PC5	PC6
Prevention	0.4402	0.0169	−0.3284	−0.4133	−0.1511	0.7102
Detection	0.4260	−0.3597	−0.2516	0.1826	0.7628	−0.1032
Response	0.4056	0.1873	0.0665	0.8225	−0.2871	0.1925
Health Capacity	0.4436	0.0048	−0.4011	−0.1815	−0.4230	−0.6561
Commitments	0.3720	−0.5276	0.7200	−0.1780	−0.1802	−0.0270
Risk Environment	0.3537	0.7462	0.3811	−0.2337	0.3185	−0.1291

PC2 explains 11.05% of the variance and has a contrasting structure: a strong positive loading on Risk Environment (0.7462) and a negative loading on Commitments (−0.5276). This captures a divide between countries’ exposure to structural/political risk and their level of formal international commitments, independent of operational capability.

The PCA results are visualised in Fig 1, with the variance explained by each component shown in Panel B, the loading heatmap in Panel C, and the PC1–PC2 biplot in Panel D.

Panel D of Fig 1 shows how countries spread along PC1, with PC2 capturing differences linked to risk exposure and commitment levels rather than to core operational capacity.

### 3.2 Bootstrap validation and component stability

The bootstrap validation produced narrow confidence intervals for PC1 loadings, with stability values ($S_{j}$) above 0.94 for all domains (Table 3). This indicates that the structure of PC1 is robust to sampling variation.

**Table 3:** Bootstrap confidence intervals for PC1 Loadings.

Domain	Mean Loading	95% CI Lower	95% CI Upper	Stability ($S_{j}$)
Prevention	0.4404	0.4278	0.4523	0.9755
Detection	0.4259	0.4133	0.4384	0.9748
Response	0.4047	0.3854	0.4214	0.9640
Health Capacity	0.4437	0.4337	0.4559	0.9778
Commitments	0.3720	0.3419	0.3956	0.9463
Risk Environment	0.3536	0.3260	0.3782	0.9478

### 3.3 Comparative analysis of resilience measures

The PCA-based resilience score was highly correlated with a simple average of domain scores (|*r*| = 0.999) and with a weighted average based on domain–GHSI correlations (|*r*| = 0.999). The absolute correlation is reported because the sign of PC1 is arbitrary; reversing the direction of the first eigenvector would change the sign but not the magnitude. While these measures align closely, the PCA score has the advantage of being grounded in the covariance structure and avoids overweighting correlated domains.

### 3.4 Country rankings and resilience distribution

The distribution of PC1 scores shows a moderate positive skew (skewness = 0.62). Table 4 lists the 15 highest-scoring countries. Traditional high-income leaders dominate the list, but it also includes upper-middle-income countries — Thailand (overall GHSI = 68.2) and Armenia (61.8) — whose 2021 GHSI performance exceeded that of many high-income peers, indicating that resilience capability, as measured here, is not a simple proxy for income level.

**Table 4:** Top 15 countries by resilience score (PC1).

Country	Resilience Score
United States	5.642
Finland	4.968
Australia	4.850
Canada	4.601
Thailand	4.460
Slovenia	4.354
United Kingdom	4.310
South Korea	4.072
Germany	4.012
Netherlands	3.939
Sweden	3.813
Denmark	3.792
New Zealand	3.531
Armenia	3.464
France	3.454

### 3.5 Regional patterns in health system resilience

Countries were grouped into five continental regions (Africa, the Americas, Asia, Europe, and Oceania) following standard UN geoscheme classifications. Because Levene’s test indicated significant heterogeneity of variance across regions (*F* = 3.71, *p* = 0.006), a Welch’s one-way ANOVA — which does not assume equal group variances — was used in place of a standard ANOVA, with Games-Howell pairwise comparisons for post-hoc testing.

Resilience scores (PC1) differed markedly across regions (Welch’s *F*[4,64.39] = 46.95, *p* < 0.001, partial $\eta^{2}=0.395$), confirming a substantial geographic gradient in health system resilience. Table 5 summarises the mean resilience scores and overall GHSI scores for each region. Europe recorded the highest mean resilience score (*M* = 2.03, *SD* = 1.54, *n* = 43; mean overall GHSI = 52.4), followed by Asia (*M* = 0.19, *SD* = 1.82, *n* = 47; overall GHSI = 40.3) and the Americas (*M* = 0.14, *SD* = 1.97, *n* = 35; overall GHSI = 39.7). Oceania (M=−1.35, *SD* = 2.23, *n* = 16; overall GHSI = 29.7) and Africa (M=−1.47, *SD* = 0.88, *n* = 54; overall GHSI = 29.1) recorded the lowest mean scores.

**Table 5:** Resilience score (PC1) and overall GHSI score by region.

Region	*n*	Mean PC1	SD PC1	Mean Overall GHSI	SD Overall GHSI
Europe	43	2.03	1.54	52.4	10.1
Asia	47	0.19	1.82	40.3	11.9
Americas	35	0.14	1.97	39.7	13.0
Oceania	16	−1.35	2.23	29.7	14.9
Africa	54	−1.47	0.88	29.1	5.8

Games-Howell post-hoc comparisons showed that Europe scored significantly higher than every other region (all *p* < 0.001). Africa scored significantly lower than the Americas, Asia, and Europe (all *p* < 0.001), but was not significantly different from Oceania (*p* = 0.999) — both regions combine a small number of high-capacity countries (e.g., Australia and New Zealand in Oceania) with a larger number of lower-capacity states, producing overlapping distributions despite different regional averages. The Americas and Asia did not differ significantly from each other (*p* = 1.000). Oceania’s wide within-region variability (*SD* = 2.23, the largest of any region, reflecting its small *n* = 16 spanning both high-income and small island states) meant its apparent gap with the Americas (*p* = 0.177) and Asia (*p* = 0.129) did not reach significance.

These regional patterns are consistent with the broader finding that resilience clusters geographically as well as structurally, reinforcing the case for region-sensitive, rather than uniform, investment strategies (Section 4.1.1).

These regional patterns are consistent with the broader finding that resilience clusters geographically as well as structurally, reinforcing the case for region-sensitive, rather than uniform, investment strategies (Section 2.16).

### 3.6 Uncertainty in country resilience estimates

Bootstrap-derived confidence intervals for country-level resilience scores averaged 0.058 in width. There was no meaningful relationship between a country’s resilience score and the precision of its estimate (r=−0.005): the 20 highest-scoring countries had a mean interval width of 0.052, and the 20 lowest-scoring countries a mean width of 0.057 — a negligible difference. Estimation precision for PC1 scores is therefore essentially uniform across the resilience distribution, rather than varying systematically with domain consistency as might be expected.

### 3.7 Regularised regression analysis

#### 3.7.1 Domain importance.

The regularised regression analysis examined the unique contribution of each domain to the overall GHSI score while addressing the strong inter-domain correlations. Table 6 compares three approaches.

**Table 6:** Comparison of domain importance measures (full-sample point estimates).

Domain	OLS Correlation	Ridge Coefficient	ElasticNet Coefficient
Prevention	0.9122	0.2182	0.2182
Detection	0.8838	0.2423	0.2420
Response	0.8185	0.1475	0.1477
Health Capacity	0.9178	0.2272	0.2270
Commitments	0.7610	0.1645	0.1644
Risk Environment	0.7270	0.1820	0.1817

The ridge results, summarised with confidence intervals in Table 7, point to Detection, Health Capacity, and Prevention as the most influential domains when controlling for overlap, with Detection showing the strongest unique contribution.

**Table 7:** Ridge coefficients with 95% bootstrap confidence intervals.

Domain	Coefficient	95% CI Lower	95% CI Upper
Prevention	0.2185	0.2055	0.2320
Detection	0.2423	0.2272	0.2563
Response	0.1471	0.1359	0.1584
Health Capacity	0.2276	0.2168	0.2394
Commitments	0.1643	0.1530	0.1783
Risk Environment	0.1817	0.1656	0.1971

*Note:* Ridge coefficients in Table 6 are point estimates from the full-sample fit; Table 7 reports bootstrap means with 95% confidence intervals. Slight differences between the two are expected due to resampling variation. The abstract cites the bootstrap means from Table 7 for consistency with reported uncertainty.

The comparison highlights three points:

1. Bivariate correlations overstate the influence of domains that are strongly correlated with others: OLS correlations range narrowly from 0.73–0.92, while ridge coefficients, which account for shared variance, show a wider and more differentiated spread (0.15–0.24).
2. Ridge and ElasticNet produce consistent results, indicating stable domain rankings.
3. Detection, Health Capacity, and Prevention emerge as the most influential domains in multivariate settings, with Detection showing the strongest unique contribution — consistent with its role as the entry point for identifying threats before they escalate.

### 3.8 Cluster analysis

The cluster analysis identified two groups of countries with a silhouette score of 0.439, indicating moderate-to-good separation. Table 8 summarises domain averages for each group.

**Table 8:** Cluster profiles: Mean domain scores and resilience characteristics.

Cluster	*n*	Prevention	Detection	Response	Health Capacity	Commitments	Risk Environment	Overall Score
Cluster 0	131	18.57	21.93	31.62	20.80	42.01	49.58	30.75
Cluster 1	64	48.66	53.40	49.78	53.38	59.71	68.66	55.60

Resilience Score (PC1): Cluster 0 = −1.23, Cluster 1 = 2.52.

Cluster 1 contains high-performing countries with strong scores across all six domains, while Cluster 0 comprises the majority of countries (67% of the sample) with lower and more structurally uneven capabilities — the gap is widest in Prevention and Health Capacity (roughly 2.6-fold) and narrowest in Risk Environment (1.4-fold), indicating that the two groups diverge most sharply on operational health-security capacity rather than on background risk exposure. Representatives of the clusters, selected as the ten highest-scoring countries by overall GHSI score within each group, are shown in Table 9.

**Table 9:** Representative countries by cluster membership.

Cluster	Representative Countries
Cluster 1	United States, Australia, Finland, Canada, Thailand, Slovenia, United Kingdom, Germany, South Korea, Sweden
Cluster 0	Vietnam, India, Jordan, Kyrgyz Republic, North Macedonia, Cyprus, Moldova, Mongolia, Costa Rica, El Salvador

**Fig 2:**
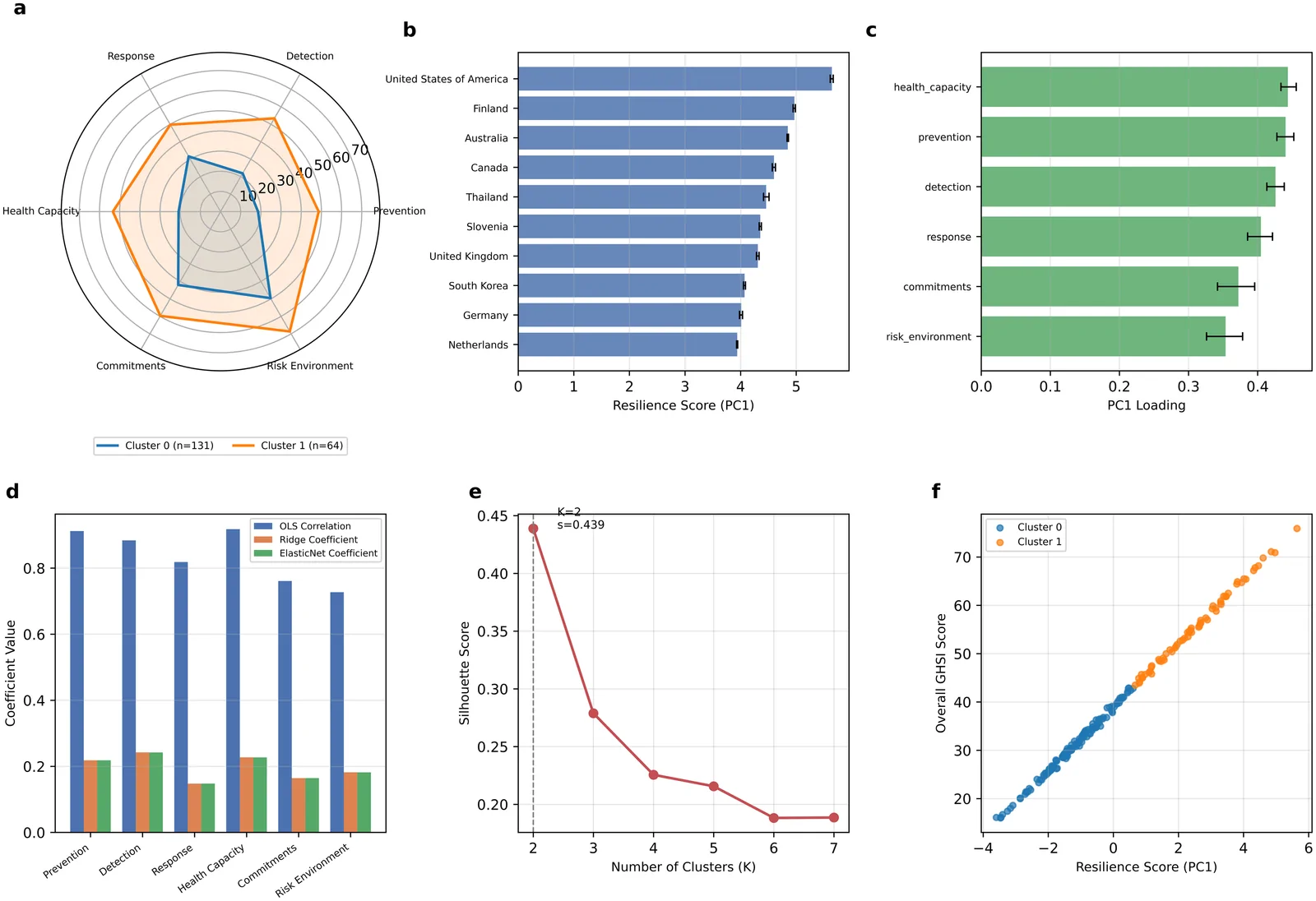
Summary dashboard of resilience analysis, including radar plots (a), top countries with confidence intervals (b), bootstrap loadings (c), domain importance (d), silhouette analysis (e), and PC1–GHSI relationship by cluster (f).

Fig 2 summarises the resilience analysis across multiple dimensions, including radar plots, domain-specific contributions, and clustering diagnostics.

## 4 Discussion

### 4.1 Policy implications for decision-makers

Our framework produces two practical outputs: (1) a ranking of resilience determinants based on their unique contributions, and (2) country typologies from cluster analysis. These outputs help move from assessment to action.

#### 4.1.1 Using typologies for tailored strategies.

The two clusters point to different starting points and different strategic paths.

**Cluster 1 (high performers, balanced capabilities, n = 64, 33% of the sample).** This group spans traditional high-income leaders (the United States, Australia, Finland, Canada, the United Kingdom, Germany, Sweden, Japan, Switzerland) together with a substantial number of upper-middle-income countries — including Brazil, China, South Africa, Mexico, Malaysia, Thailand, Turkey, Indonesia, and Argentina — whose 2021 GHSI performance places them in the same capability profile despite lower income levels. This confirms that strong, balanced health security capacity is achievable outside the traditional high-income group, and that Cluster 1 membership reflects investment and institutional performance rather than income status alone. Their focus should shift from building capacity to sustaining and integrating systems. Priorities include:

- Maintain investments across domains while improving coordination among surveillance, response, and health care delivery.
- Invest in advanced preparedness (digital surveillance, genomic sequencing, rapid countermeasure development).
- Build agility to handle overlapping crises (e.g., pandemics with climate events or conflicts).
- Support global health security through technical assistance and regional capacity building — the upper-middle-income members of this cluster (e.g., Brazil, South Africa, Thailand) are natural anchors for South–South technical cooperation, given their demonstrated capacity and regional proximity to lower-performing neighbours.

**Cluster 0 (uneven capabilities, specific gaps, n = 131, 67% of the sample).** This group includes most low- and lower-middle-income countries — among them Nigeria, Pakistan, Bangladesh, India, Vietnam, Ghana, Kenya, and Egypt — together with several higher-scoring members (e.g., Vietnam, Jordan, Mongolia, Costa Rica) whose overall profile remains structurally uneven relative to Cluster 1. The gap between clusters is widest in Prevention (2.6-fold) and Health Capacity (2.6-fold), and narrowest in Risk Environment (1.4-fold) — indicating that operational health-security capacity, not background risk exposure, is what most separates the two groups. The framework recommends targeted, sequenced investments:

- Start with detection systems and core health functions – these yield the largest resilience gains.
- Use phased strategies: first address laboratory capacity, early warning systems, and basic infrastructure; then move to higher-order capabilities, with Prevention and Health Capacity as the priority gap areas given the size of the between-cluster divide.
- Form technical partnerships with Cluster 1 countries — including higher-capacity peers within their own region or income group — to accelerate knowledge transfer.
- Pursue regional collaboration to share resources (e.g., reference laboratories, joint training, shared stockpiles).

#### 4.1.2 Prioritising investments by domain importance.

Our ridge regression (descriptive, not causal) shows that Detection, Health Capacity, and Prevention contribute most to the overall GHSI score after accounting for correlations, with Detection showing the single largest unique contribution (ridge coefficient = 0.242, 95% CI: 0.227–0.256). This hierarchy can guide budget allocation.

For national health planners:

1. **Detection first**: strengthen laboratory networks, surveillance, and early warning. Timely threat identification enables effective response, and Detection’s leading ridge coefficient confirms it as the domain with the strongest independent association with overall performance once shared variance with the other five domains is accounted for.
2. **Health capacity as the platform**: invest in workforce, infrastructure, and service models that support routine care and surge capacity (ridge coefficient = 0.228).
3. **Prevention as a multiplier**: support public health programmes that lower baseline disease burden and free resources for emergencies (ridge coefficient = 0.219).
4. **Enabling conditions for sustainability**: address governance, financing, and risk factors that affect long-term performance — Risk Environment (0.182) and Commitments (0.164) contribute meaningfully but less than the top three domains.

For donor agencies:

- Design funding that prioritises detection and health capacity while also supporting the enabling environment.
- Create metrics to track progress across the priority domains.
- Tailor technical assistance to the specific gaps identified in each cluster.
- Encourage South-South and triangular cooperation based on similar capability profiles, not just geographic proximity — the presence of Brazil, South Africa, Thailand, and Mexico in Cluster 1 provides concrete anchor countries for this model.

#### 4.1.3 Practical implementation steps.

We propose a three-step pathway:

1. **Assess and benchmark**: calculate the resilience score (PC1), identify cluster membership, and compare with peers.
2. **Gap analysis**: use the domain importance rankings to find specific weaknesses in detection, health capacity, and prevention.
3. **Strategy and resource mobilisation**: develop investment plans with clear sequencing and allocate resources accordingly.

Because the framework is transparent and reproducible, countries can track progress over time and adjust strategies based on measurable outcomes.

#### 4.1.4 Limitations in implementation.

Several points deserve attention. Our analysis measures structural capacity, not real-time crisis performance. Policymakers should supplement these findings with simulation exercises and after-action reviews. Contextual factors (political stability, community trust, informal care networks) may also affect resilience beyond what the GHSI captures. Finally, the cross-sectional design cannot capture dynamic changes or path dependencies.

Despite these caveats, the framework provides a robust, evidence-based starting point. It answers both *what* to prioritise and *how* to tailor strategies to different country contexts.

#### 4.1.5 Situating findings within the broader resilience literature.

Our typology and domain-importance findings can be read alongside two strands of existing work: studies that apply similar data-driven methods to the GHSI, and the substantial post-COVID-19 literature questioning the GHSI’s predictive validity.

**Convergence with related PCA/clustering analyses.** Nasser et al. (2025) applied PCA and multi-scenario K-means clustering to the 2017–2021 GHSI, restricted to high-income countries and using the 37 underlying indicators rather than the six published domain scores. They identified nine components explaining 74.5% of total variance, with the first and dominant component (“Foundational Capacity, Regulations, Resilience, and Prevention”) loading most heavily on prevention- and capacity-related indicators [24]. This is broadly consonant with our single dominant PC1 (70.6% of variance), which loads similarly across Prevention, Health Capacity, and Detection. The difference in component count, one dominant axis in our analysis versus nine in theirs, is attributable to methodological scope rather than contradictory structure: restricting the sample to high-income countries and analysing indicator-level rather than domain-level data increases the effective dimensionality that PCA must resolve. Both studies converge on the same substantive conclusion: prevention- and capacity-oriented domains carry the largest share of shared variance in GHSI-based resilience measures. Our finding that two-cluster typologies separate countries primarily by *integration across domains* rather than by excellence in any single domain also echoes Kruk et al.’s (2017) conceptual framework, in which an “integrated” health system, one that coordinates capacities across domains rather than concentrating them narrowly, is proposed as a defining characteristic of resilience, independent of the country’s income level [2].

**Divergence and a necessary caution: GHSI’s contested predictive validity.** A substantial body of work published after the 2019 GHSI edition found that raw GHSI scores did not predict, and in some analyses were associated in the opposite direction with, actual COVID-19 outcomes. Aitken et al. (2020) reported that higher GHSI scores were associated with *higher*, not lower, COVID-19 burden across the countries examined [25]. Haider et al. (2020) found no significant correlation between GHSI or Joint External Evaluation scores and countries’ COVID-19 detection response time or mortality outcomes [26]. Abbey et al. (2020) reached a similar conclusion restricted to OECD countries [27]. Boyd, Wilson and Nelson (2020) offered a more mixed picture, finding strong criterion validity of the GHSI against the Joint External Evaluation ReadyScore but only moderate association with communicable-disease mortality more broadly [28]. More recently, Stoto, Nelson and Kraemer (2023) mounted a methodological defence of preparedness indices, arguing that the outcome measures used in these critiques, typically cumulative case or death counts at a single time point, are themselves too simplistic to fairly evaluate a multidimensional, cross-sectional preparedness construct against a highly heterogeneous, multi-wave pandemic [29].

This literature matters directly for how our findings should be interpreted. Our PCA-derived resilience score and cluster typology are built entirely from the GHSI’s own six domain scores; they are a data-driven *summary and reweighting* of the GHSI’s internal structure, not an independently validated predictor of real-world pandemic outcomes. Where the domain-importance and typology results in this study describe how the GHSI’s own domains relate to one another and to its overall score, they should not be read as claims about which domains would have mattered most for COVID-19 mortality specifically, a question the literature above shows the GHSI itself has not reliably answered. We consider this an important boundary condition for our policy recommendations (Section 2.16.2) rather than a reason to discount them: the ranking of Detection, Health Capacity, and Prevention reflects which domains are least redundant with one another *within* the GHSI’s construction, which remains useful for interpreting and prioritising within the index even if the index’s absolute scores are an imperfect predictor of outcomes in any single pandemic. We return to this distinction in Limitations.

### 4.2 Limitations and future research

This study has several limitations that inform future work:

- The GHSI domains and overall score are mathematically linked (*R*^2^ = 1.00 in the ridge regression, by construction), limiting causal inference. Our regression results are descriptive only and should be read as a decomposition of the GHSI’s built-in weighting structure rather than as evidence of causal influence.
- Because the resilience score and domain-importance rankings are derived entirely from the GHSI’s own structure, they inherit any limitations in the GHSI’s construct validity. Several COVID-19-era studies found that raw GHSI scores were poor predictors of, or even associated in the opposite direction with, actual pandemic outcomes [25–27], though this remains contested on methodological grounds [29]. Our findings should therefore be read as a characterisation of the GHSI’s internal structure and relative domain weighting, not as a validated predictor of future pandemic performance.
- The cross-sectional data do not allow analysis of how resilience evolves over time.
- The cluster separation (silhouette score 0.439) indicates moderate-to-good but not complete separation; countries near the cluster boundary should be interpreted as occupying a continuum rather than belonging to discrete, non-overlapping groups.
- The GHSI captures structural capacity, not real-time performance. Political and governance factors are only partly measured.
- Regional analysis (Section 2.12) used a coarse five-continent classification; a finer-grained regional or income-group stratification, and time-series data spanning multiple GHSI editions, would allow a more precise test of geographic and economic patterning.
- Nonlinear relationships and threshold effects may exist and warrant investigation.

## 5 Conclusion

This study presents a structured, statistically grounded approach to measuring health system resilience using PCA, bootstrap validation, regularised regression, and cluster analysis, applied to the complete 2021 Global Health Security Index dataset (195 countries). The first principal component explains 70.6% of the variance across six GHSI domains, and the loadings are highly stable (bootstrap stability $S_{j}>0.94$ for all domains). Regularised regression (descriptive) shows that detection, health capacity, and prevention contribute most to the overall index after accounting for multicollinearity, with detection showing the strongest unique contribution. Two distinct country clusters emerge: one with strong, balanced capabilities (n = 64, 33% of countries) and another with lower, more structurally uneven capabilities (n = 131, 67%) — a division driven primarily by gaps in Prevention and Health Capacity rather than in background risk environment, and one that cuts across income groups rather than tracking them directly.

These findings reinforce the view that resilience depends on coordinated development across detection, prevention, health sector capacity, and enabling conditions. As global health threats evolve, the framework offers a practical foundation for tracking progress and prioritising investments to build stronger, more responsive health systems.

## Supporting information

S1 Data Processed dataset containing domain scores, first principal component (PC1) resilience scores, and cluster assignments for all 195 countries in the 2021 Global Health Security Index. Derived from the raw 2021 GHSI dataset (https://www.ghsindex.org/).
(XLSX)

S1 Code Python scripts implementing the complete reproducible analysis pipeline (00_prepare_data.py through 07_build_S1_data.py), which regenerates every table, figure, and the S1 Data file from the raw public GHSI source. (ZIP)
